# Unsupervised learning using EHR and census data to identify distinct subphenotypes of newly diagnosed hypertension patients

**DOI:** 10.1371/journal.pone.0326776

**Published:** 2025-07-09

**Authors:** Jaclyn M. Hall, Jie Xu, Marta G. Walsh, Hee-Deok Cho, Grant Harrell, Shailina A. Keshwani, Steven M. Smith, Stephanie A. S. Staras

**Affiliations:** 1 Department of Health Outcomes & Biomedical Informatics, University of Florida, Gainesville, Florida, United States of America; 2 Department of Pharmaceutical Outcomes & Policy, University of Florida, Gainesville, Florida, United States of America; 3 Department of Community Health & Family Medicine, University of Florida, Gainesville, Florida, United States of America; 4 Division of Cardiovascular Medicine, Department of Medicine, University of Florida, Gainesville, Florida, United States of America; University of North carolina at Greensboro, UNITED STATES OF AMERICA

## Abstract

**Background:**

Hypertension (HTN) is a complex condition with significant heterogeneity in presentation and treatment response. Identifying distinct subphenotypes of HTN may improve our understanding of its underlying mechanisms and guide more precise treatment or public health initiatives.

**Methods:**

Using EHR and Medicaid claims data from the OneFlorida+ research consortium (2012–2021), we identified a cohort of adult Floridians with newly diagnosed HTN (first diagnosis following two outpatient blood pressures ≥140/90 mmHg & no prior anti-HTN treatment). We extracted demographic and clinical data from the diagnosis visit and ≤1 year prior. We used hierarchical clustering (unsupervised machine learning) to identify distinct subphenotypes within the OneFlorida+ HTN population.

**Results:**

A total of 40,686 patients were included (mean ± SD age, 60.9 ± 17.5 y; 55% women). Five subphenotypes (S1-5) were identified. S1 was characterized by older age, higher Body Mass Index (BMI), and prevalent type 2 diabetes. S2 included over 50% of Black patients who were primarily women, younger, with higher BMI, but living in communities with higher levels of socioeconomic vulnerabilities. S3 contained a higher percentage of Hispanic patients with comparatively lower BMI. S4 is characterized by higher age and co-morbidities. S5 had 94% of patients with chronic kidney disease. Distinctions in social determinants of health factors were also observed.

**Conclusions:**

Unsupervised learning identified 5 HTN subphenotypes varying in demographic, socioeconomic, and risk profiles. Further investigation into the biological mechanisms of these subphenotypes and the relationships to social factors may enhance our ability to deliver targeted interventions that consider social policy implications in addition to the traditional behavioral and physiological interventions.

Controlling high blood pressure (BP) is paramount to public health. High BP, also known as hypertension (HTN) is a serious circulatory condition that afflicts approximately 48% of U.S. adults [1]. HTN is the leading underlying risk factor contributing to death and morbidity worldwide. Despite several evidence-based guidelines and proven interventions, only 1 in 4 U.S. adults have their BP under control [1]. In the US, cardiovascular health (CVH) remains concerningly low, and there are multiple opportunities to monitor, maintain, and improve CVH in individuals and the population [2].

HTN can affect people of all ages, genders, and ethnic backgrounds. Certain factors can increase the risk of developing HTN, and these include advancing age, gender, family history of HTN, smoking, obesity, and chronic conditions, including kidney disease, diabetes, and sleep apnea [3–6]. For example, men are more likely to smoke than women but also more likely to be physically active [2,7]. Thus, tailored interventions to focus on reducing smoking may be needed for subgroups of men. It is also well established that some ethnic groups have a higher predisposition for developing HTN, including individuals of African, Caribbean, or South Asian descent [8–11]. Tailored interventions, based on a group’s cultural values that reflect the behavioral preferences and expectations of the group, are more successful [12].

Successful hypertension management is strongly influenced by social context, like education and income [13]. Incorporating known non-medical drivers of health can improve the accuracy of hypertension risk models by accounting for environmental and social factors that contribute to disease development and progression. Many environmental and community factors—like chronic stress from substandard housing or financial insecurity—can raise blood pressure, worsen control and contribute to related conditions such as poor sleep and mental health [14–16]. Hypertension disproportionately affects marginalized groups and community measured social determinants of health, such as poverty or limited access to care, contribute to disparities in hypertension outcomes [17]. Community level variables provide the critical context that helps explain the higher risk faced by some patients, despite their clinical profiles, leading to improved risk stratification and more targeted interventions. A better understanding of the contribution of these social determinants of health can inform policymakers and health systems seeking to align resources with the needs of the community, e.g., community clinics, transportation services, or education campaigns in underserved areas.

Despite the knowledge that HTN is a complex condition with significant heterogeneity in presentation and treatment response [3,18], standard clinical recommendations to improve CVH suggest a one-size-fits-all treatment approach that includes an extensive list of behavioral and clinical interventions. The clinically supported recommendation by the American Heart Association Life’s Essential 8 ™ is to improve CVH by focusing on eight categories: 1) manage cholesterol, 2) control blood sugar, 3) lower BP, 4) improve nutrition, 5) increase physical exercise, 6) manage a healthy weight, 7) refrain from smoking, and 8) sleep 7–9 hours daily [19]. While critical to CVH, only a small fraction (0.45%) of the U.S. population is reaching optimal goals for all eight measures [2]. Large differences exist in the scores; thus, the health interventions needed to achieve these eight measures will also differ across groups [20].

Asking a patient with, or at risk for, HTN to undergo multiple health interventions simultaneously can potentially be overwhelming and challenging for them to manage effectively. Implementing multiple interventions simultaneously might be unrealistic for some patients, leading to willpower depletion, behavioral inertia, or the natural resistance to change a habit. These factors are amplified when patients have limited time, resources, or support [21]. To successfully improve their odds of preventing HTN, clinicians could guide patients in beginning their journey to better health by focusing on one or two of the eight components (e.g., nutrition and physical exercise). There is evidence that the choice of the priority intervention matters and may differ by population subgroups. For example, because racial/ethnic disparities in HTN prevalence are significant, identifying the most beneficial targeted interventions may be predicted by the needs and circumstances of population subgroups [22,23]. However, there is little evidence to assist clinicians in determining which areas of change should be prioritized.

By recognizing and categorizing HTN subphenotypes, we can potentially tailor more precise and effective treatments, leading to better patient outcomes. Additionally, identifying distinct subphenotypes of HTN may advance our understanding of the underlying mechanisms of this condition. In this paper, we applied hierarchical clustering to analyze patient data to identify distinct subgroups of patients with HTN. This allowed for a detailed exploration of the variations within the hypertensive population and provided insights that could enhance our approach to managing HTN.

## Methodology

### Ethics statement

This study was conducted using anonymized HIPAA-limited electronic health records obtained from the OneFlorida+ Data Trust. The OneFlorida+ Data Trust Consortium reviewed and approved the approach and variables. The study data were fully de-identified and did not contain any personal identifiers that could be linked to individual patients, ensuring the protection of patient privacy. This retrospective study did not require direct interaction with human subjects. The study was conducted in full compliance with relevant ethical standards for human data research and was approved by the University of Florida Institutional Review Board (IRB# IRB202201071).

### Data source and study population

This study utilized EHR and Medicaid claims data from the OneFlorida+ Clinical Research Network (CRN) [24], covering from January 1, 2012 to December 31, 2021, and data were received in August of 2022. OneFlorida+ is a comprehensive clinical research network with a longitudinal dataset of over 17 million Floridians’ real-world patient-level data, encompassing Medicaid claims, cancer registries, vital statistics, and EHRs from clinical partners (health systems with broad geographic coverage across the state). The network complies with the PCORnet Common Data Model, providing anonymized information on patient demographics, vital signs, conditions, encounters, diagnoses, procedures, prescriptions, dispensing, and laboratory results.

**Fig 1:**
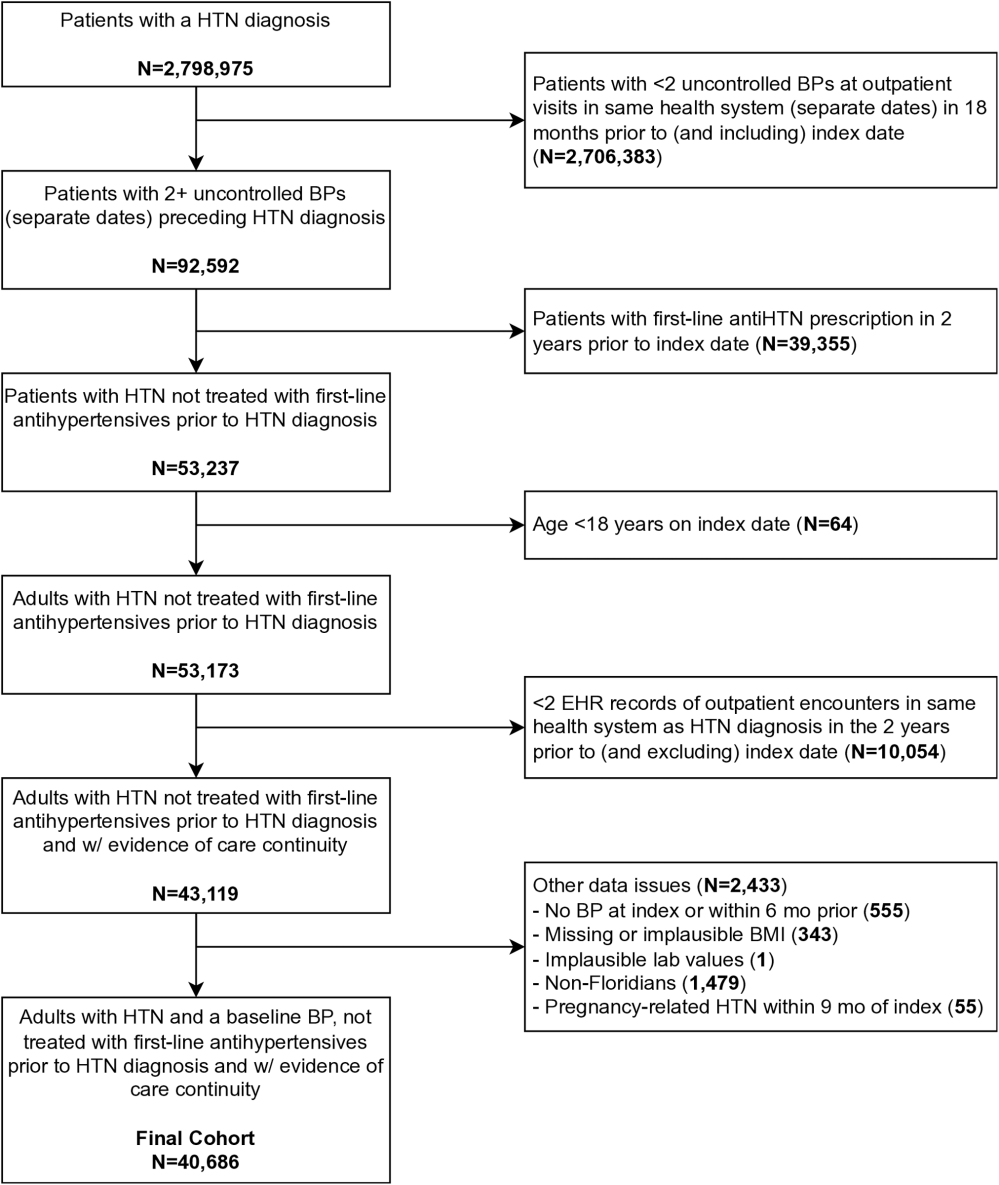
Flow diagram for development of initial HTN diagnosis cohort.

To identify the study population, we applied a strict criterion to develop the base cohort of patients with newly diagnosed HTN, similar to our prior studies [25]. Specifically, we included adult individuals (age ≥ 18 years) who received their first HTN diagnosis, marked by ICD-9 codes 401.x or ICD-10 codes I10-I15, with the first HTN diagnosis being considered the *index date*. To ensure the accuracy and reliability of our findings, and avoid patients with missing data, we identified patients who are meaningfully engaged with the healthcare system. Patients were required to have ≥2 prior elevated outpatient BP readings on separate dates from the same health system within the 18 months preceding (and inclusive of) the index date. Elevated BP was defined as a systolic BP ≥ 140 or diastolic BP ≥ 90 mm Hg. Patients were excluded if they had a prior history of prescribed 1^st^-line antihypertensive treatment (angiotensin-converting enzyme inhibitor [ACEI], angiotensin receptor blocker [ARB], calcium channel blocker [CCB], thiazide diuretic, or β-blocker). Additionally, to maximize the inclusion of consistent patients (who would be expected to have the most relevant healthcare data at the health system in which they were determined to have incident HTN), we required that patients have ≥2 outpatient encounters over the 24 months preceding (and exclusive of) the index date, at the same health system as the elevated BP and HTN diagnosis. Finally, we excluded non-Florida residents, those without a BP in the 6 months prior to and including the index date, those with missing or physiologically implausible lab or other biometric measurements, and those with pregnancy-associated HTN (ICD-10 O12-O16 or ICD-9 642.3–642.6 within nine months of index date) (Fig 1). The study protocol received ethical approval from the Institutional Review Board of the study institution.

### Data preprocessing

In this study, the clustering analysis utilized essential demographic and clinical information, such as comorbidities and prescribed drugs, from the diagnosis visit and the period up to one year before the index date. A total of 41 variables from OneFlorida+ EHR included five demographic variables (gender, age, race, ethnicity, insurance type), a history of 17 comorbidities, 21 clinically relevant variables, 13 lab results, BMI, and the Charlson Comorbidity Score (CCS). The CCS is a well-validated index incorporating 19 categories of medical conditions identifiable in medical records, allowing for a more precise understanding of the burden of chronic disease at a population level [26]. In addition, to gain insights into the contextual factors influencing patient health outcomes, patient’s 5-digit residential zip codes were used to link to community-level factors associated with the social determinants of health. We identified the Zip Code Tabulation Area (ZCTA) for each 5-digit zip code. ZCTAs are the generalized spatial representations of the geographic extent of the mail routes that a ZIP Code represents, built using Census blocks. We linked each patient’s most recent residential location 5-digit zip code to 17 variables related to the Social Determinants of Health (SDoH) from Census.gov. These included ZCTA level unemployment rate, percent poverty, percentage of population without a high school degree, etc.). Including community variables enabled us to consider broader social and environmental determinants that might impact HTN, providing valuable context for our research.

Thorough data preprocessing, described below, was conducted to ensure the quality and reliability of the dataset used for HTN subphenotyping. For variables with missing values, we employed imputation techniques using the median value of that specific variable among all patients with values. Additionally, we handled categorical variables like race and binary attributes like sex, using one-hot encoding [27] to transform categorical variables into numerical representations, facilitating their incorporation into our hierarchical clustering analyses.

### Subphenotyping with agglomerative hierarchical clustering

**Fig 2:**
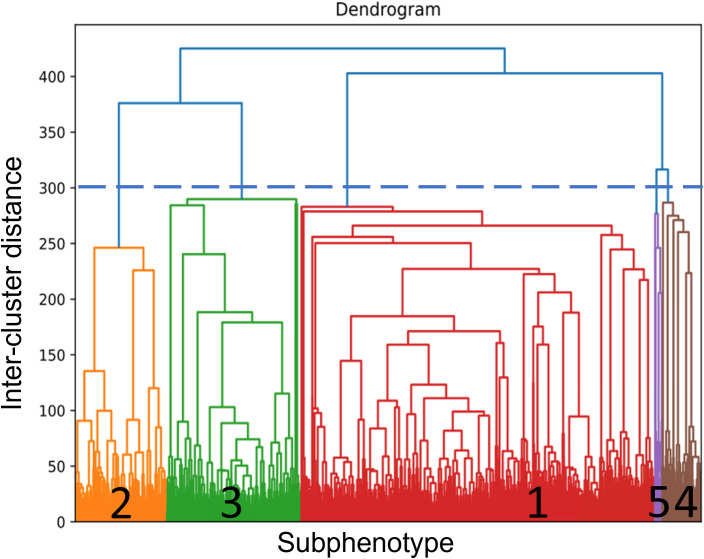
Dendrogram depicting the five selected subphenotypes of HTN patients. The blue dotted line represents the cut-point, or the initial differentiation of the clustering outcome. The horizontal axis represents the inter-cluster distances, while the vertical axis represents the distribution of patients with HTN.

Hierarchical cluster analysis aims to group objects or records that are “close” to one another, and the calculation of distance measures is repeated between clusters, which are then grouped into larger clusters. The outcome is represented graphically as a dendrogram. Following the preprocessing of variables as previously described, we employed agglomerative hierarchical clustering of the cohort using Ward linkage to derive subphenotypes [28]. The primary objective was to minimize within-group dispersion at each binary fusion step. The linkage function specifies the distance between two clusters and is computed as the increase in the “error sum of squares” (ESS) after combining patients from the two clusters into a single cluster. It is repeated until only two clusters remain. The resulting dendrogram initially differentiated the clustering outcomes, illustrated in Fig 2. To identify the optimal number of clusters that best captured the underlying patterns within the data, we utilized two distinct metrics, i.e., the gap statistic to compare the change in within-cluster dispersion with that expected under an appropriate reference null distribution [29], and Davies-Bouldin index to evaluate the ratio between the cluster scatter and the cluster separations [30].

After completing the clustering process, we conducted a series of statistical tests to explore potential differences among the identified subphenotypes. For categorical variables, we applied the chi-square test [31]. In the case of continuous data, we first assessed the normality of the variables using the Shapiro-Wilk test [32]. For those variables found to be normally distributed, we performed adjustments for age and sex using One-Way ANOVA analysis [33]. Additionally, we applied the Kruskal-Wallis test for variables exhibiting a skewed distribution [34]. This approach to clustering and subsequent statistical analysis aimed to reveal meaningful patterns and associations within the data, providing insights into the characteristics of the distinct HTN subphenotypes.

## Results

Between January 1, 2012, and June 30, 2021, 2,798,975 patients with an HTN diagnosis were recorded in OneFlorida + . We identified a unique cohort of 40,686 adult Floridians with newly diagnosed HTN (first diagnosis following two outpatient BPs ≥ 140/90 mmHg and no prior anti-HTN treatment) who remained within the same health system. Among this final cohort, 55% were female, 44% were 45–64 years old, 20% were Black, 22% were Hispanic, and 0.08% had no race or ethnicity identified (Table 1).

**Table 1:** Newly diagnosed HTN Patient Cohort from OneFlorida (2012–2021).

		Male n (%)	Female n (%)	Total
	HTN Patient Cohort	18,328 (45%)	22,358 (54%)	40,686
Age (years)	18-44	2,876 (45%)	3,577 (55%)	6,453
	45-64	7,873 (44%)	9,895 (56%)	17,768
	>=65	7,556 (46%)	8,879 (54%)	16,435
Race	Native Amer./Alaskan	26 (59%)	18 (41%)	44
	Native Hawaiian or Pacific Islander	160 (42%)	220 (58%)	380
	Asian	2,903 (36%)	5,144 (63%)	8,047
	Multiple Race	13,990 (47%)	15,494 (53%)	29,484
	Black American	199 (49%)	204 (51%)	403
	White	1 (12%)	7 (87%)	8
	Unknown/other	1,049 (45%)	1,271 (55%)	2,320
Ethnicity	Hispanic	4,237 (47%)	4,863 (53%)	9,100
	Non-Hispanic	13,569 (45%)	16,904 (55%)	30,473
	Unknown	522 (47%)	591 (53%)	1,113

The average age of the final cohort was 60.9 (± SD 17.5) years, with an average BMI of 30.5 kg/m^2^ and 23% with records of current tobacco use (Table 2). The average combined comorbidity score for the entire cohort was 1.3. Only 6.1% of the patients were insured by Medicaid, 30% were insured by Medicare, and 45% were privately insured. Also of note- 10% of the cohort had a history of depression, 15% had a history of sleep disorder, 15% also had a history of type 2 diabetes, and 26% had a history of chronic kidney disease. The mean LDL-cholesterol was in the normal range for the entire cohort, but the mean value for the protective HDL-cholesterol, 51.5 mg/dL, was lower than the recommended ≥60 mg/dL.

**Table 2:** Characteristics of 5 HTN subphenotypes derived from unsupervised hierarchical clustering. Clustering was guided by the Davies-Bouldin index and Gap Statistics to determine the appropriate number of clusters. Cells are color-scaled for each individual row where the maximum value is red, the minimum value is deepest blue, the mean of the values would be white, and cells are colored based on the value’s relative position between minimum and maximum. Ordinary least squared regression p-values, adjusting for age and sex. All values < .05 unless indicated by a * (* denotes p-value > .05).

		Total	S1	S2	S3	S4	S5	Adj. ols (p-value)
	**Variables**	**40686**	**N = 22991**	**N = 5983**	**N = 8712**	**N = 2518**	**N = 482**
	Female	22358 (0.54)	12806 (0.55)	3719 (0.62)	4507 (0.51)	1128 (0.44)	198 (0.41)	0.00
Race	Native American or Alaskan	44 (0)	44 (0)	0 (0)	0 (0)	0 (0)	0 (0)	0.00
	Native Hawaiian or Pacific Islander	8 (0)	0 (0)	0 (0)	8 (0)	0 (0)	0 (0)	0.00
	Asian	380 (0)	3 (0)	0 (0)	375 (0.04)	1 (0)	1 (0)	0.00
	Multiple Race	403 (0)	1 (0)	0 (0)	389 (0.04)	5 (0)	8 (0.01)	0.00
	Black or African American	8047 (0.19)	3971 (0.17)	3122 (0.52)	345 (0.03)	432 (0.17)	177 (0.36)	0.00
	White	29484 (0.72)	17742 (0.77)	2648 (0.44)	6848 (0.78)	1969 (0.78)	277 (0.57)	0.00
Ethn.	Hispanic	9100 (0.22)	1136 (0.04)	1976 (0.33)	5415 (0.62)	451 (0.17)	122 (0.25)	0.00
	Not Hispanic	30473 (0.74)	21276 (0.92)	3810 (0.63)	3022 (0.34)	2010 (0.79)	355 (0.73)	0.00
Payer	Private	18454 (0.45)	11448 (0.49)	2435 (0.4)	3824 (0.43)	644 (0.25)	103 (0.21)	0.00
	Medicaid	2480 (0.06)	553 (0.02)	1405 (0.23)	192 (0.02)	265 (0.1)	65 (0.13)	0.00
	Medicare	12081 (0.29)	7440 (0.32)	1000 (0.16)	2189 (0.25)	1219 (0.48)	233 (0.48)	0.00
	Government other	713 (0.01)	684 (0.02)	0 (0)	9 (0)	13 (0)	7 (0.01)	0.00
	Current/Recent Smoking	9431 (0.23)	5680 (0.24)	1367 (0.22)	1389 (0.15)	897 (0.35)	98 (0.2)	0.00
History of	Anticoagulant Use	1953 (0.04)	327 (0.01)	10 (0)	1322 (0.15)	267 (0.1)	27 (0.05)	0.00
	Aspirin Use	560 (0.01)	12 (0)	0 (0)	14 (0)	516 (0.2)	18 (0.03)	0.00
	Statin Use	1331 (0.03)	965 (0.04)	47 (0)	22 (0)	282 (0.11)	15 (0.03)	0.02
	Coronary Heart Disease	1426 (0.03)	95 (0)	0 (0)	17 (0)	1263 (0.5)	51 (0.1)	0.00
	Stroke or TIA	231 (0)	4 (0)	0 (0)	0 (0)	223 (0.08)	4 (0)	0.00
	ASCVD	2191 (0.05)	129 (0)	0 (0)	27 (0)	1954 (0.77)	81 (0.16)	0.00
	Atrial Fibrillation*	1212 (0.02)	833 (0.03)	1 (0)	11 (0)	343 (0.13)	24 (0.04)	0.16
	HF with reduced EF	343 (0)	6 (0)	0 (0)	0 (0)	306 (0.12)	31 (0.06)	0.00
	Peripheral Arterial Disease	746 (0.01)	37 (0)	0 (0)	10 (0)	660 (0.26)	39 (0.08)	0.00
	Percut. Coronary Interv.	208 (0)	2 (0)	0 (0)	0 (0)	205 (0.08)	1 (0)	0.00
	COPD*	1090 (0.02)	792 (0.03)	5 (0)	8 (0)	271 (0.1)	14 (0.02)	0.26
	Asthma	1503 (0.03)	1347 (0.05)	5 (0)	25 (0)	115 (0.04)	11 (0.02)	0.00
	Depression	4054 (0.09)	2791 (0.12)	481 (0.08)	364 (0.04)	394 (0.15)	24 (0.04)	0.00
	Sleep Disorder (non-OSA)	6237 (0.15)	4070 (0.17)	592 (0.09)	1018 (0.11)	513 (0.2)	44 (0.09)	0.00
	Obstructive Sleep Apnea	1138 (0.02)	925 (0.04)	9 (0)	9 (0)	179 (0.07)	16 (0.03)	0.00
	Type 2 Diabetes	6195 (0.15)	3290 (0.14)	961 (0.16)	1005 (0.11)	759 (0.3)	180 (0.37)	0.00
	Gout	542 (0.01)	483 (0.02)	0 (0)	6 (0)	32 (0.01)	21 (0.04)	0.00
	Chronic Kidney Disease	10607 (0.26)	7612 (0.33)	973 (0.16)	738 (0.08)	827 (0.32)	457 (0.94)	0.00
	End-stage Renal Disease	345 (0)	0 (0)	0 (0)	2 (0)	4 (0)	339 (0.7)	0.00
	Kidney Transplant	162 (0)	0 (0)	0 (0)	0 (0)	0 (0)	162 (0.33)	0.00
		**Mean (Variance)**	**S1**	**S2**	**S3**	**S4**	**S5**	
	Age	60.9 (17.5)	17.6 (60.4)	16.3 (56.6)	16.8 (62.5)	17.7 (70.4)	15.6 (59.5)	0.00
	BMI	30.5 (7.6)	8 (30.7)	7.7 (31.7)	6.2 (29.3)	7.2 (29.3)	6.7 (28.2)	0.00
	Combined Comorbidity	1.3 (2.8)	2.6 (1.2)	2.2 (0.8)	2.8 (1.2)	3.6 (3.6)	3.1 (5.3)	0.00
Value	Serum Creatinine	0.9 (0.4)	0.2 (0.9)	0.1 (0.9)	0.1 (0.9)	0.3 (0.9)	2.8 (2.2)	0.00
	Serum Potassium	4.2 (0.3)	0.3 (4.2)	0.2 (4.2)	0.1 (4.2)	0.3 (4.2)	0.4 (4.3)	0.00
	Uric Acid*	5.7 (0.3)	0.3 (5.7)	0 (5.7)	0.1 (5.7)	0.1 (5.7)	0.7 (5.8)	0.88
	Estimated GFR	80.7 (12.6)	14 (80.9)	9.2 (82.2)	6.1 (81.1)	13.9 (77.3)	29.8 (62.2)	0.00
	Diastolic BP (mmHg)	82.4 (11.5)	11.3 (83.1)	11.1 (83.6)	11.7 (81.2)	11.2 (77.8)	12.9 (79.3)	0.00
	HDL-Cholesterol	51.5 (8.5)	10.5 (51.8)	6.1 (51.4)	2.2 (51)	7.7 (50.9)	7.7 (51)	0.00
	LDL-Cholesterol	108.6 (17.4)	20.5 (109.2)	14.1 (108.7)	5 (108)	19.6 (105.4)	17.6 (104.1)	0.00
	Total Cholesterol	190.6 (19.9)	24 (191.4)	14.7 (190)	5.8 (189.8)	20.6 (187.6)	21.7 (186)	0.00
	Alanine Transaminase	20.7 (15.8)	20.5 (21.8)	5.1 (19.3)	3.1 (19.2)	7.6 (19.5)	10.6 (19.1)	0.00
	Aspartate Transferase	21.5 (16.3)	21.2 (22.4)	4.9 (20.3)	2.7 (20.1)	7.5 (21.1)	9 (20.3)	0.00
	Triglycerides	120.4 (53.3)	67.9 (123.9)	23.1 (115.1)	9.9 (115.3)	38.6 (119.4)	44.8 (121.3)	0.00
	Systolic BP (mmHg)	144.4 (16.8)	16.9 (144.4)	16.4 (144.5)	15.8 (144.5)	18.6 (142.5)	21.1 (147.1)	0.00
	Hemoglobin A1c	5.9 (2.4)	0.7 (5.9)	0.6 (5.9)	0.3 (5.8)	0.7 (5.9)	21.1 (6.8)	0.04
ZCTA	disability – civilian	13 (4.7)	4.6 (13.7)	4.8 (14.5)	3.2 (10)	4.8 (14.1)	4.3 (12.8)	0.00
	persons below poverty	14.9 (8.1)	7.9 (13.8)	8.1 (22.6)	5 (12.3)	8.4 (15.6)	8.3 (16.5)	0.00
	unemployment Rate	5.7 (2.6)	2.4 (5.5)	3 (7.8)	1.8 (4.7)	2.5 (5.9)	2.8 (6.3)	0.02
	persons aged <= 17	20.2 (4.9)	5.1 (19.9)	4.6 (22.1)	4.6 (19.7)	4.9 (20.1)	5.1 (20.6)	0.00
	single parent with children	36.8 (14.4)	12.8 (34.2)	14.3 (52.1)	10.9 (32.5)	14.8 (38.2)	14.7 (40.3)	0.00
	no vehicle available	7.3 (5.9)	3.9 (5.8)	7.9 (13.3)	5.8 (6.9)	6.3 (7.7)	6.8 (8.7)	0.00
	no HS diploma (age 25+)	12.6 (7.7)	5.9 (10.3)	7.6 (21.7)	7 (12.2)	8 (13.2)	8.5 (14.6)	0.00
	Overcrowding	3.4 (2.6)	1.7 (2.4)	3.3 (5.7)	2.6 (4.3)	2.6 (3.4)	2.9 (4.1)	0.00
	Minority (non NH white)	53.5 (28.1)	23 (40.6)	22.5 (79.6)	22 (70)	28.2 (50.5)	27.9 (61.2)	0.00
	speak Eng. “less than well”	13.9 (15)	9.2 (7.7)	20.4 (22.6)	13.8 (24.5)	15.5 (12.3)	15.1 (17)	0.00
	Structures with>= 10 units	19.5 (19.6)	15.8 (15.2)	18.2 (20.4)	24.3 (30.5)	20.2 (17.8)	19.9 (21.1)	0.00
	mobile homes	9.4 (14)	15.3 (12.1)	13.1 (8.1)	5.4 (2.4)	15.8 (12.2)	11.5 (7.6)	0.00
	per capita income$	31,273 (13,849)	32,432 (13,359)	20,407 (5,946)	36,195 (15,482)	29,975 (12,221)	28,703 (12,046)	0.30
	median family income $	57,834 (20,593)	59115 (17911)	40,266 (12,160)	67,535 (24,291)	55,115 (19,305)	53,695 (19,579)	0.00
	median gross rent $	1,216 (373)	1,144 (317)	1,044 (253)	1,549 (387)	1,133 (351)	1,222 (372)	0.00
	Employed in white-collar	6.4 (2.7)	6.9 (2.9)	4.6 (2)	6.1 (1.9)	6.2 (2.7)	6.1 (2.5)	0.00
	persons aged 65 and older	18.3 (9.5)	19.3 (11.2)	16 (4.5)	17.2 (6.3)	18.8 (8.7)	18.1 (9.1)	0.00

Five subphenotypes of new HTN patients were identified by evaluating the optimal clustering statistics, with patient numbers ranging from 482 to 22,991 (Table 2). Colors represent the highest (red) and lowest (blue) values, with shading indicating where values fall within the range of each row. Characteristics of each contributing variable to the subphenotypes are given in Table 2, including the adjusted p-value result of testing the statistical differences between subphenotypes. Only three clinical variables (history of atrial fibrillation, COPD, and uric acid measurement) and one community-level variable (per capita income) did not show statistical differences across subphenotypes. Two race variables had < 50 individuals—Native American/Alaskan and Native Hawaiian/Other Pacific Islander.

Subphenotype 1 had the largest membership (22,991 patients, 57%) and had a characterization more closely reflecting the overall cohort for demographic and most clinical variables. Compared to the other subphenotypes, Subphenotype 1 has higher participation in private insurance, a higher EHR recorded history of asthma, depression, and sleep disorders, and higher lab values for cholesterol, triglycerides, ALT, and AST. Patients in S1 also tended to live in ZCTAs with communities associated with less socioeconomic vulnerability and less minority population but a higher percentage of mobile homes.

Subphenotype 2 (5,983 patients, 15%) had a larger share of female (62%) and Black patients (52%) compared with the overall cohort. S2 members were characterized by the youngest mean age, the least comorbidities (mean CCS = 0.8), the highest percent with Medicaid insurance, higher BMI, and a higher percent of members residing in ZCTAs with the socioeconomic burden (e.g., higher poverty, unemployment, a person with a disability, adults with less than high school education).

Subphenotype 3 (8,712 patients, 21%) had a higher percentage of White and Hispanic patients than the overall cohort. Most Asian and multi-race patients were members of S3. S3 had relatively low rates of comorbidities (mean CCS = 1.2), a low mean BMI, and a comparatively lower smoking rate (16%). S3 members also resided more frequently in communities with higher incomes and less vulnerability and minority populations who speak English ‘less well.’

Subphenotype 4 (2,518 patients, 6%) had a higher percent White and non-Hispanic patients. This group represents older patients, mostly with Medicare insurance, 36% of which smoke, and who have multiple comorbidities (mean CCS = 3.6), including a high percent with history of various cardiac-related conditions (50% with coronary heart disease, 77% with atherosclerotic heart disease, and 9% with history of stroke). This subphenotype was associated with a higher percentage of residences in ZCTAs that closely aligned with the overall cohort on social and economic variables.

Subphenotype 5 (482 patients, 1%) was a small, specific group comprised of individuals with chronic kidney disease (95%), end-stage kidney failure (70%), and the highest levels of creatine, potassium, and uric acid. S5 had the second lowest mean age and the lowest BMI but the highest mean CCS (5.3). SDoH variables showed only slightly more burden than the larger population.

## Discussion

We identified five subphenotypes of patients with a first diagnosis of HTN. Derived from an EHR and claims data trust representing over 17 million Floridians, our results represent those Floridians seeking and receiving health care. While the majority of the patients were separated into a more traditional risk factor group of older White adults with some chronic conditions and Black patients with obesity, we also identified a group consisting of older Hispanics and two distinct groups of patients with more advanced chronic conditions. These subphenotypes represent a first step in the process of precision prioritization of the Essential Eight to specific groups of patients, which may lead to more effective and efficient control of BP.

Our overall cohort had expected differences compared to those reported for the Florida population. First, similar to other EHR-based studies, the percentage of females is higher than that of males, as women seek health care services more than men. Additionally, for HTN specifically, older women have a higher risk for HTN after losing the beneficial effects of hormones post-menopause [35,36]. Second, the racial and ethnic breakdown of the overall cohort of newly diagnosed HTN aligns with that of the population of the state of Florida in higher age categories [37]. While the HTN cohort has a lower percentage of Hispanics compared to Florida, this is expected because of the lower prevalence of HTN in US Hispanic populations and the smaller (but growing) proportion of older-age Hispanics in Florida [38]. Third, consistent with HTN risk factors of higher smoking rates (cohort = 23.2% vs. Florida = 14.7%) and diabetes (cohort = 15.2% and Florida = 11.8%) [37].

Our results demonstrated significant differences in clinical values and cooccurring condition presentation across different subphenotypes. Subphenotype 1 contained 56.5% of the initial newly HTN diagnosed cohort and represents what might be thought of as a historically typical patient with HTN: majority White, non-Hispanic, ~ 60 years of age, overweight with high cholesterol, having a history of sleep-disordered or depression, at risk of kidney disease, and about 1 in 4 are tobacco users. This subphenotype may benefit from interventions around maintaining weight, lowering cholesterol, and maximizing restful sleep. Members of S1 were from various communities but were more likely to live in a community of mobile homes, possibly representing residents of the many rural areas or wintering retiree communities in Florida. In contrast, Subphenotype 2 had a higher share of Black patients and women, lower mean age at HTN onset, and fewer comorbidities, yet higher mean BMI. Both obesity and HTN affect Black men and women at younger ages in the US [39]. Patients in this subphenotype are more likely to live in communities with higher poverty, a higher percentage of non-White residents, and have a lower income [40]. This subphenotype is an important target group, and clinicians can prioritize these younger Black patients for early identification of HTN to prevent further HTN-related complications. Subphenotype 3 represents Florida’s first sizeable population of retiring Hispanic residents. This group is slightly higher in mean age than the entire HTN cohort. Still, on average, they are relatively healthy and tend to live in communities with higher income, possibly representing the large Hispanic population in South Florida. This subphenotype may benefit most from maintaining weight and other healthy lifestyle behaviors. Almost all Asians in the cohort were placed in Subphenotype 3, demonstrating how our clustering method efficiently identifies even small subgroups. Subphenotype 4 has a higher age and much higher rates of comorbidities. This subphenotype of primarily White men represents an important group that needs concentrated efforts to boost cardiovascular wellness, along with help with sleep disorders, depression, tobacco use, and diabetes. They also live in communities with higher rates of residents living in mobile homes.

Americans from a broad spectrum of community types struggle to achieve optimal CVH [41]. In their 1988 report, the Joint National Committee on Detection, Evaluation, and Treatment of High Blood Pressure recommended considering patient demographic characteristics when selecting initial treatment options [42]. The standard treatments for HTN have evolved as new drugs have become available and with new understandings of health measures associated with improving and maintaining CVH [19, p. 8]. Still, patients face unique challenges in managing CVH, including maintaining healthy behaviors and utilizing preventative health services [43]. With the exception of per-capita income, our five subphenotypes differ significantly in SDoH and demographic factors. Our results demonstrated the significant differences in clinical values and cooccurring condition presentation across different subphenotypes.

Considering sociodemographics and factors related to patient residential community will be necessary to produce both culturally relevant and clinically effective health interventions [44]. Interventions should include health education strategies tailored to specific groups and target a broader set of locations for public health engagement, such as mobile home communities and community centers is low income areas, as the subphenotypes outline. For example, men may have higher prevalence of hypertension earlier in life, and lower hypertension awareness [45]. Thus, men with a profile aligning with Subphenotype 1 may benefit from early monitoring and interventions that address occupational stress or dietary habits common in male populations.

### Strengths and limitations

The authors acknowledge several limitations and strengths of this study. The study relied on EHRs and Medicaid claims data, which may not capture all relevant patient data [46]. However, using the OneFlorida+ Data trust, which takes multiple approaches to improve data quality [24], we are assured that data have been standardized across different health data providers. The data used were only from the state of Florida. Florida has the third largest population and is one of the most representative states, as it closely aligns with the nation’s population on matters of race, education, income, employment, etc. [47]. Therefore, studies using data from Florida can be considered generalizable. In addition, OneFlorida+ has EHR and claims data for 16 million FL patients at the time of this study [24], and the final cohort of newly diagnosed HTN patients was sufficiently large to conduct population-level studies. We chose a well-established and interpretable method to identify preliminary patient subgroups that may have clinical relevance. As an exploratory effort, this study offers a starting point for future investigations that could employ more advanced or tailored clustering approaches to further examine these patterns. By incorporating SDoH-related data, we complemented the traditional understanding of clinical patient types. Some variables may contain larger proportions of missing data than others. While we minimized the impact of missingness by employing standard imputation techniques, we acknowledge the missing data may not be random. For laboratory measurements (e.g., ALT and AST, both measures of liver function, or lipid measurements of HDLC, LDLC, and TRIG), the missingness may be correlated. However, the variables are interesting because the values are informative and represent modifiable health measures.

## Conclusions

We employed machine learning to delve beyond classic risk factors for hypertension. We identified distinctly different demographic populations that may exhibit different risks and ultimately benefit from different intervention strategies through precision public health initiatives. Unsupervised learning identified 5 HTN subphenotypes varying in demographic, socioeconomic, and risk profiles. These subtypes inform our understanding of HTN patients and the potential barriers they may face to controlling their BP. Identifying distinct subphenotypes of HTN may improve our understanding of the underlying mechanisms of HTN and guide the development of personalized and precise treatment of individuals. Further investigation into the biological mechanisms of these subphenotypes could reveal their potential and barriers to successful blood pressure control. This greater understanding will enhance our ability to deliver targeted interventions that consider social policy implications in addition to the traditional behavioral and physiological interventions.
